# Automated Quantitative CD8+ Tumor-Infiltrating Lymphocytes and Tumor Mutation Burden as Independent Biomarkers in Melanoma Patients Receiving Front-Line Anti-PD-1 Immunotherapy

**DOI:** 10.1093/oncolo/oyae054

**Published:** 2024-04-24

**Authors:** Dylan Fortman, Arivarasan Karunamurthy, Douglas Hartman, Hong Wang, Lindsey Seigh, Ibrahim Abukhiran, Yana G Najjar, Liron Pantanowitz, Hassane M Zarour, John M Kirkwood, Diwakar Davar

**Affiliations:** Department of Medicine, University of Pittsburgh Medical Center, Pittsburgh, PA, USA; Department of Dermatology, University of Pittsburgh and UPMC, Pittsburgh, PA, USA; Department of Pathology, University of Pittsburgh and UPMC, Pittsburgh, PA, USA; Department of Pathology, University of Pittsburgh and UPMC, Pittsburgh, PA, USA; Department of Biostatistics, University of Pittsburgh and University of Pittsburgh Medical Center, Pittsburgh, PA, USA; Department of Pathology, University of Pittsburgh and UPMC, Pittsburgh, PA, USA; Department of Pathology, University of Pittsburgh and UPMC, Pittsburgh, PA, USA; Division of Hematology-Oncology, Department of Medicine, UPMC Hillman Cancer Center, Pittsburgh, PA, USA; Department of Pathology, University of Michigan, Ann Arbor, MI, USA; Division of Hematology-Oncology, Department of Medicine, UPMC Hillman Cancer Center, Pittsburgh, PA, USA; Division of Hematology-Oncology, Department of Medicine, UPMC Hillman Cancer Center, Pittsburgh, PA, USA; Division of Hematology-Oncology, Department of Medicine, UPMC Hillman Cancer Center, Pittsburgh, PA, USA

**Keywords:** melanoma, metastatic, TIL, tumor mutation burden, TMB/Mb, immunotherapy, biomarker, PD-1, PD-L1

## Abstract

**Background:**

CD8+ tumor-infiltrating lymphocyte (TIL) predicts response to anti-PD-(L)1 therapy. However, there remains no standardized method to assess CD8+ TIL in melanoma, and developing a specific, cost-effective, reproducible, and clinically actionable biomarker to anti-PD-(L)1 remains elusive. We report on the development of automatic CD8+ TIL density quantification via whole slide image (WSI) analysis in advanced melanoma patients treated with front-line anti-PD-1 blockade, and correlation immunotherapy response.

**Methods:**

Seventy-eight patients treated with PD-1 inhibitors in the front-line setting between January 2015 and May 2023 at the University of Pittsburgh Cancer Institute were included. CD8+ TIL density was quantified using an image analysis algorithm on digitized WSI. Targeted next-generation sequencing (NGS) was performed to determine tumor mutation burden (TMB) in a subset of 62 patients. ROC curves were used to determine biomarker cutoffs and response to therapy. Correlation between CD8+ TIL density and TMB cutoffs and response to therapy was studied.

**Results:**

Higher CD8+ TIL density was significantly associated with improved response to front-line anti-PD-1 across all time points measured. CD8+ TIL density ≥222.9 cells/mm^2^ reliably segregated responders and non-responders to front-line anti-PD-1 therapy regardless of when response was measured. In a multivariate analysis, patients with CD8+ TIL density exceeding cutoff had significantly improved PFS with a trend toward improved OS. Similarly, increasing TMB was associated with improved response to anti-PD-1, and a cutoff of 14.70 Mut/Mb was associated with improved odds of response. The correlation between TMB and CD8+ TIL density was low, suggesting that each represented independent predictive biomarkers of response.

**Conclusions:**

An automatic digital analysis algorithm provides a standardized method to quantify CD8+ TIL density, which predicts response to front-line anti-PD-1 therapy. CD8+ TIL density and TMB are independent predictors of response to anti-PD-1 blockade.

**Implications for Practice**

Developing a specific, cost-effective, reproducible, and clinically actionable biomarker to anti- programmed death 1 (PD)-(L)1 blockade has remained elusive. This article reports on the development of digital whole-slide imaging for automated CD8+ TIL density quantification, which in this study was shown to be both feasible and an independent predictive biomarker of response to anti-PD-1 therapy in patients with advanced melanoma.

## Background

Programmed death 1 (PD-1) is a receptor expressed by activated T and B cells which binds to PD-L1 (B7-H1)^1,2^ and PD-L2 (B7-DC).^3,4^ PD-1 negatively regulates T-cell functions through the engagement of PD-L1, which is expressed by a wide variety of tissues.^1-4^ In tumors, tumor-antigen (TA)-specific CD8+ T cells exhibit a diminished capability to produce cytokines, proliferate and eliminate tumor cells in a progressive and hierarchical fashion.^5-7^ TA-specific T cells express PD-1 and other inhibitory receptors^6,8^; consequently, anti-PD(L)1 blockade with monoclonal antibodies increases the number of TA-specific CD8+ T cells and enhances their lytic function preclinically^9^ and in ex vivo melanoma.^5^ In advanced melanoma, anti-PD-(L)1 blockade with immune checkpoint inhibitors (ICI) is associated with objective responses in 35%-40% of patients^10,11^ with 35% progression free at 1 year^12^ and 34% alive at 5 years.^13^

Multiple biomarkers of response to anti-PD-(L)1 blockade across tumors have been described including CD8+ tumor-infiltrating lymphocytes (TIL),^14-16^ PD-L1 expression,^15,17,18^ tumor mutation burden (TMB),^19-21^ changes in exhausted-phenotype CD8+ T cells peripherally^22,23^ and CD8+ T-cell clonotypic expansion intra-tumorally and peripherally.^24-26^ However, developing a specific, cost-effective, reproducible, and clinically actionable biomarker to anti-PD-(L)1 blockade remains elusive. While quantitative and semi-quantitative immunohistochemical (IHC) assessment of CD8+ TIL in melanoma has been reported in several studies^17,27^; there remains no standardized method to assess CD8+ TIL and hence, each study analyzed CD8+ TIL differently. Several studies have attempted quantification using density (ie, number of CD8+ T cells per area of measurement)^28^; and one study used digital pathology software with machine learning algorithms to link CD8+ TIL density to targeted therapy response.^29^

Herein, we report the development of an image analysis algorithm that automatically quantified CD8+ TIL density in whole slide images acquired from stained tissue sections from advanced melanoma patients treated with front-line anti-PD-(L)1 blockade. Correlation between automated CD8+ TIL density and immunotherapy outcomes are analyzed and reported. Further, analysis of automated CD8+ TIL density and TMB as assessed using targeted NGS revealed both to be independent predictors of response to front-line anti-PD-(L)1 blockade.

## Methods

### Patient Selection

Approval was obtained from the University of Pittsburgh Cancer Institute (UPCI) Institutional Review Board (IRB) for a retrospective tissue analysis of patients with advanced melanoma who received treatment with front-line single agent anti-PD-1 (IRB number PRO18080253). Tissue specimens were retrieved from the Department of Pathology’s biorepository. Seventy-eight patients treated with either nivolumab, pembrolizumab, or investigational PD-1 inhibitors between January 2015 and May 2023 in the front-line setting were included in this analysis. Of these, 61 patients had tumors that were additionally molecularly profiled by targeted NGS. A prespecified sample size was not determined. Response Evaluation Criteria in Solid Tumors (RECIST) version 1.1 was used to assess efficacy; and radiographic response was assessed by the treating investigator (D.D., Y.N., or J.M.K.). Patients who were not radiologically evaluable were excluded from this analysis. Progression-free survival (PFS) was defined as time from immunotherapy start date to date of radiographic or clinical progression. Patients who had not progressed were censored at the date of their last follow up. Overall survival (OS) was calculated from immunotherapy start date to date of death; and patients who were alive were censored at the date of last follow-up.

### Immunohistochemistry

Four micrometer tissue sections from representative tissue blocks were sectioned and mounted on positively charged glass slides. The tissue blocks selected were obtained prior to any systemic therapy had been administered. CD8+ IHC was performed using the C8/144B clone (catalog number GA62361-2; Agilent Dako, Santa Clara, CA). Pretreatment of tissue was performed, and antibody was diluted to a concentration of 1:40. DAB (3,3ʹ-diaminobenzidine) was used as the color reagent for visualization. Sections of human tumor known to contain abundant CD8+ lymphocytes were used as positive control. A monoclonal rabbit immunoglobulin G isotype antibody (catalog no. 760-1029; Roche Diagnostics) was applied to replicate samples as a negative reagent control.

### Digital Quantitative Image Analysis

Following CD8 staining, whole slide images were digitized using an Aperio AT2 (Leica Biosystems, Wetzlar, Germany) scanner at 40× magnification. Once uploaded in eSlideManager (eSM), digital slide was de-arrayed and manually adjusted to ensure that entire stained section was completely within individual de-array image. The entire tumor was manually annotated on the IHC digital slide after review of the associated H&E digital slide by an experienced pathologist. A previously validated nuclear image analysis algorithm (Leica Biosystems, Wetzlar, Germany) was used to count and threshold CD8-positive cells within the tumor annotation. All positive cells were included in the results irrespective of the staining intensity (weak, moderate, or strong staining). CD8 positive cells in areas away from the tumor (outside the tumor annotation) were excluded. The CD8 TIL density was calculated using the following equation: CD8 TIL density = total CD8-positive cells within tumor annotation (cells)/ area of entire tumor annotation (mm^2^).

### TMB quantification

Targeted next-generation sequencing (NGS) was performed using Oncomine V3 (0.29Mb) assay. Briefly, DNA was extracted from tumors. Bar-coded libraries were generated and sequenced for targeted hotspots, exons and select promoter regions and introns of a gene panel of 161 genes (Supplementary Table S1). Massive parallel sequencing was carried out on an Ion S5 XL System according to the manufacturer’s instructions (Thermo Fisher Scientific, Waltham, MA) and data were analyzed with the Torrent Suite Software v5.8.17 and an in-house bioinformatics program, Variant Explorer (UPMC) for point mutations, small insertions/deletions and copy number alterations. Each variant was prioritized according to the 2017 Association for Molecular Pathology/American Society of Clinical Oncology/College of American Pathologists joint consensus guidelines for interpretation of sequence variants in cancer using a tier-based system.^30^ The minimum depth of coverage for testing was 300×. For each mutation identified, an AF was calculated based on the number of reads of the mutant allele versus the wild-type allele and reported as a percentage. The limit of detection of the assay for single-nucleotide variants/indels was at 5% mutant allele frequency (AF); however, only mutations over 10% allelic frequency is used for TMB calculation. The variants that have AF in range of 45%-55% are also excluded due to possibility of being germline. UPMC Oncomine TMB formula compensated for the small panel size and used a weighted approach for inclusion of variants. The criteria for inclusion of a variant in TMB calculation included variant classification, genomic location, allelic fraction, and minor allele frequency in population databases.

### Statistical Analysis

The relationship between markers (CD8+ TIL density and TMB) and clinical response was studied by comparing the marker values between responders and non-responders, using the 2-sided 2-sample *t*-test. Before doing these tests, we checked if the data were normally distributed. If not, then we sought a Box-Cox power transformation to normalize the data, using the maximum likelihood procedure in SAS PROC TRANSREG (SAS Institute Inc., Cary, NC). For CD8 density and MSK percentile, we identified 0.25 and 1.25 as a reasonable parameter for power transformation, respectively. For TMB/Mb, we found a log transformation was reasonable for the *t*-test. For markers with significant association with 3-month response, we found cutoff points based on the ROC curves, and evaluated the performance of these cutoff points by fitting univariate logistic regression models for the response outcomes. For PFS and OS, Kaplan-Meier survival curves were plotted in subgroups. The relationship between each marker and survival endpoints (PFS and OS) was analyzed with the Cox proportional hazards models.

## Results

### Patients and Clinical Characteristics

Seventy-eight patients who received front-line single-agent anti-PD-1 therapy were included. Demographic information is provided in Table 1. The patients, 49 males (62.8%) and 29 females (37.2%), were aged 21-88 years (median age, 68.5 years). Most patients were of Caucasian ethnicity (*n* = 76, 97.4%). According to the American Joint Committee on Cancer (AJCC) eighth edition melanoma classification, the largest number of patients had M1c disease (*n* = 25, 32.1%) while a subset of patients had IIIB/C unresectable (*n* = 13, 16.7%), M1a (*n* = 19, 24.4%), M1b (*n* = 11, 14.1%), or M1d disease (*n* = 10, 12.8%). Most patients had received no prior treatment (*n* = 74, 94.9%) prior to anti-PD-1 therapy. Of these 78 patients, 39 (50%) received nivolumab, 38 (48.7%) received pembrolizumab, and 1 (1.3%) received an investigational anti-PD-1 agent. The majority of samples were from the skin or lymph node specimens (78%) (Table 2).

**Table 1.** Patient characteristics.

**Number of patients**	78
Age (years) at start of therapy Median (range)	68.5 (21-88)
**Sex**	
Male	49 (62.8%)
Female	29 (37.2%)
**Race**	
Caucasian	76 (97.4%)
Non-Caucasian	2 (2.6%)
**Extent of disease at start of therapy (AJCC eighth edition)**	
IIIB/C-unresectable	13 (16.7%)
M1a	19 (24.4%)
M1b	11 (14.1%)
M1c	25 (32.1%)
M1d	10 (12.8%)
**Lactate dehydrogenase (LDH) level (U/mL)**	
Normal	13 (16.7%)
1-2× upper limit of normal (ULN)	53 (67.9%)
>2× ULN	12 (15.4%)
**Prior adjuvant therapies**	
None	74 (94.9%)
Anti-CTLA-4	3 (3.8%)
IFN	1 (1.3%)
**Anti-PD-1 therapy**	
Nivolumab	39 (50.0%)
Pembrolizumab	38 (48.7%)
Investigational anti-PD-1	1 (1.3%)
**Source of biopsy**	
Skin/LN	61 (78.2%)
Lung	8 (10.3%)
Non-brain visceral	6 (7.7%)
Brain	3 (3.8%)

**Table 2.** CD8+ TIL density and TMB by biopsy source

	CD8+ TIL density		TMB	
Group	Mean (±SD)	*N* (%)	Mean (±SD)	*N* (%)
Skin/LN	145.4 (±213.7)	61 (78.2%)	16.7 (±25.2)	47 (77.1%)
Lung	161.0 (±152.3)	8 (10.3%)	30.4 (±16.2)	6 (9.8%)
Non-lung visceral including skeletal	119.1 (±160.3)	6 (7.7%)	24.7 (±15.5)	5 (8.2%)
Brain	277.8 (±377.9)	3 (3.8%)	15.3 (±7.3)	3 (4.9%)

To compare CD8+ TIL density and TMB by biopsy source, the data were summarized as above, and an ANOVA *F*-test performed. The ANOVA *F*-test *P*-value had a non-significant *P*-value of .7362 (WSI CD8+ TIL density comparison) and .5346 (TMB comparison), indicating that the 4 groups did not have significant differences, and accordingly post hoc Tukey’s tests were deemed unnecessary.

### Correlation Between CD8+ TIL Density and Response to Anti-PD-1 Immunotherapy

**Figure 1.**
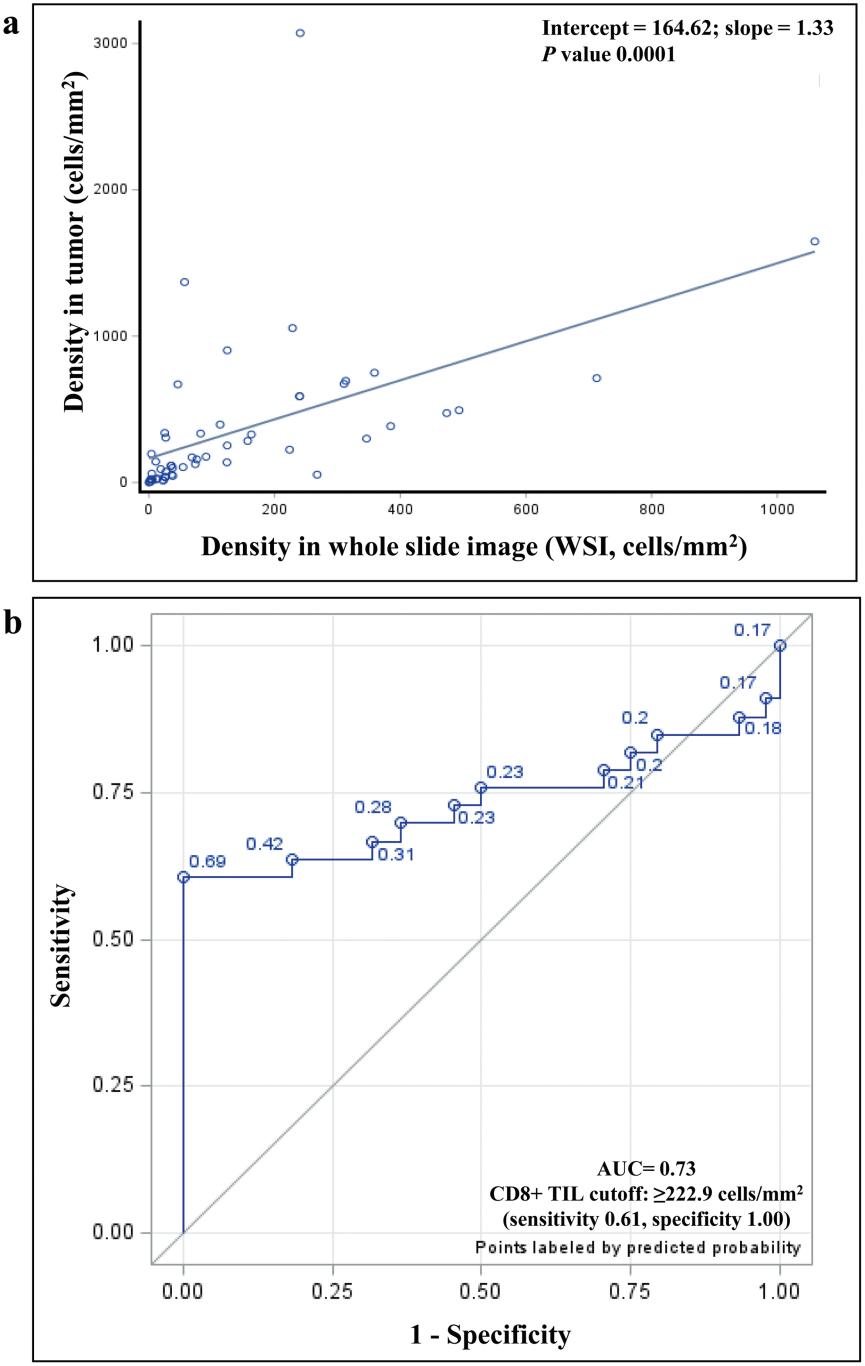
Correlation between WSI and tumor CD8+ TIL infiltrate in PD-1 treated melanoma patients, and derivation of 222.9 cells/mm^2^ Cutoff.

CD8+ TIL was quantitated in pretreatment tumor tissue as outlined in Methods. The algorithmically defined whole slide image (WSI) CD8+ TIL density was tightly correlated with the manually defined CD8+ TIL (Fig. 1A; *P* = .0001), and hereafter used for all analyses. The WSI CD8+ TIL density did not significantly differ by the biopsy source site (ANOVA F-test *P*-value .7362) (Table 2). Higher WSI CD8+ TIL density was significantly associated with improved objective response (ORR) to anti-PD-1 therapy across time points measured (3-month ORR, 6-month ORR) and with both best response and current response achieved (Table 3) with one patient currently receiving ongoing therapy. In order to generate a WSI CD8+ TIL density cutoff, we used the receiver operative curve (ROC) method which identified a CD8+ TIL density cutoff of ≥222.9 cells/mm^2^ that reliably segregated responders and non-responders (sensitivity 0.61, specificity 1.00, AUC 0.73) (Fig. 1B). Herein, in patients with CD8+ TIL density ≥222.9 cells/mm^2^, the observed 3-month ORR was 100% (20/20, 95% CI 83% - 100%), and commensurate 3-month ORR in patients with CD8+ TIL that was <222.9 cells/mm^2^ was 22.8% (13/57, 95% CI 13%-36%; *P* < .0001). Similarly, the observed 6-month ORR in patients with CD8+ TIL density ≥222.9 cells/mm^2^ was 94.7% (18/19, 95% CI 74.0%-99.9%), and commensurate 6-month ORR in patients with CD8+ TIL that was <222.9 cells/mm^2^ was 21.1% (12/57, 95% CI 11.4%-33.9%) (*P* < .0001). Hence, the CD8+ TIL density cutoff of ≥222.9 cells/mm^2^ reliably separated responders from non-responders to anti-PD-1 therapy regardless of when response was measured.

**Table 3.** Association of CD8+ TIL density and response to therapy.

	Responders		Non-responders		*P*-value (2 sample *T* test)
	Mean (±SD)	*N*	Mean (±SD)	*N*	
3-month ORR	270.8 (±272.3)	33	59.6 (±51.1)	44	.0009
6-month ORR	260.6 (±268.3)	30	72.6 (±108.9)	46	.0024
Best response	248.0 (±264.6)	32	73.2 (±110.8)	44	.0046
Current response	277.8 (±291.6)	22	93.5 (±133.7)	54	.0011

### Correlation Between CD8+ TIL Density and PFS and OS to Anti-PD-1 Therapy

**Figure 2.**
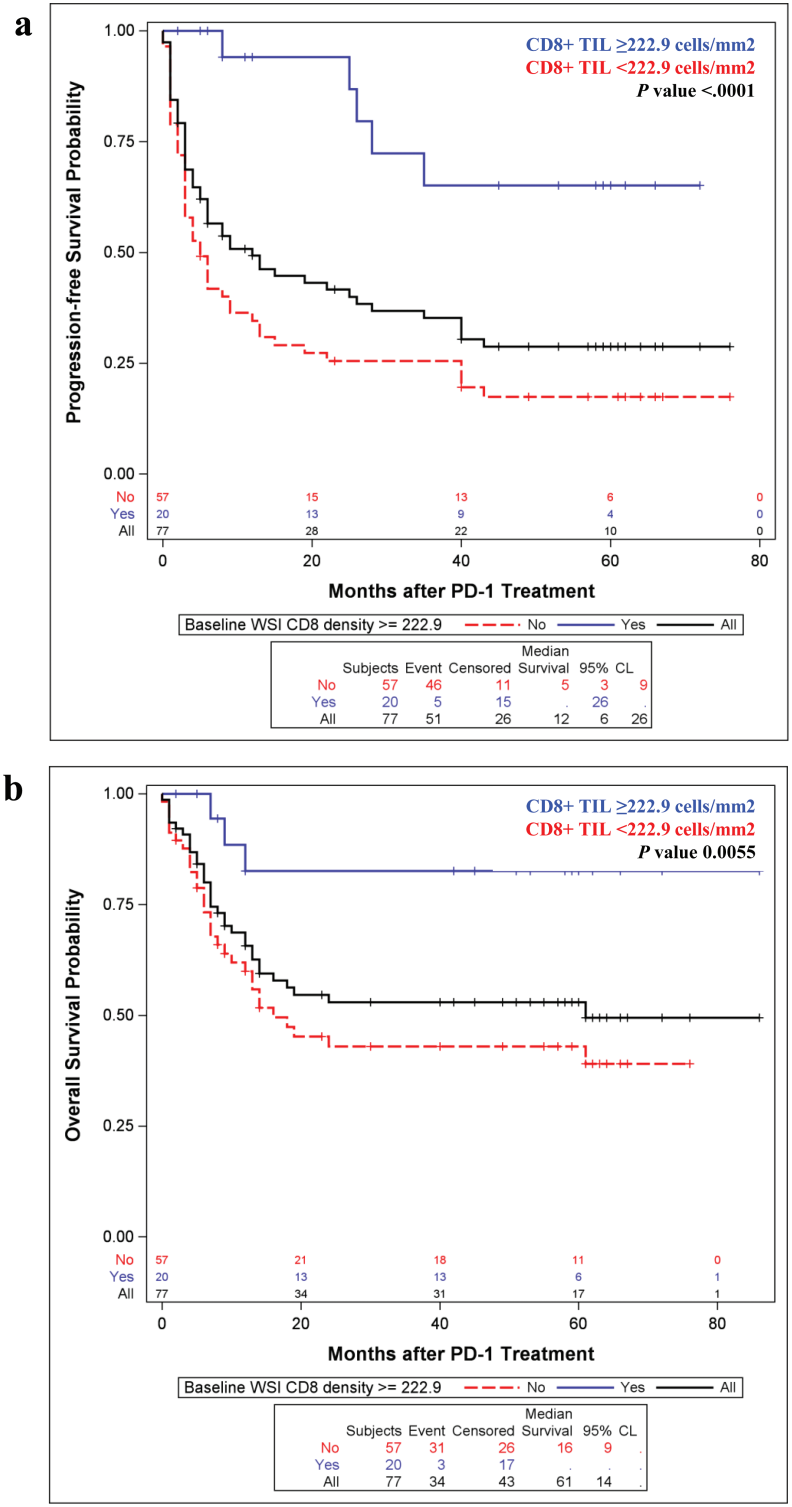
Relationship between elevated CD8+ TIL and PFS and OS in PD-1 treated melanoma.

Increased CD8+ TIL density was associated with improved PFS and OS in melanoma patients treated with anti-PD-1 immunotherapy. Median PFS in patients with CD8+ TIL density ≥222.9 cells/mm^2^ significantly exceeded those with CD8+ TIL density <222.9 cells/mm^2^ (not reached vs. 5 months, *P* < .0001; Fig. 2A). Additionally, median OS in patients with CD8+ TIL density ≥222.9 cells/mm^2^ significantly exceeded those with CD8+ TIL density <222.9 cells/mm^2^ (not reached vs. 16 months, *P* = .0055, Fig. 2B). When controlling for age, gender, extent of disease (binary variable of stage M1 vs. stage III-unresectable disease) and lactate dehydrogenase levels (LDH), patients with CD8+ TIL density ≥222.9 cells/mm^2^ continued to have significantly greater PFS than those with CD8+ TIL density <222.9 cells/mm^2^ (*P* = .0086) with a trend toward improved OS (*P* = .0844; Table 4).

**Table 4.** Multivariate analysis of CD8+ TIL infiltrate, TMB/Mb, and response to therapy.

Biomarker	Hazard ratio	95% CI	*P*-value
*Progression-free survival*			
Age (continuous variable)	1.01	0.98, 1.03	.5800
Female gender	0.66	0.32, 1.37	.2679
Stage (M1 vs. unresectable III)	1.33	0.56. 3.13	.5194
Elevated LDH (LDH ≥upper limit of normal)	1.002	1.001, 1.003	.0004
Elevated CD8+ TIL ≥222.9 cells/mm^2^ vs. <222.9 cells/mm^2^	0.24	0.08, 0.69	.0086
Elevated TMB ≥14.70 Mut/Mb vs. <14.70 Mut/Mb	0.42	0.20, 0.88	.0209
*Overall survival*			
Age (continuous variable)	1.02	0.99, 1.06	.1278
Female gender	1.10	0.44, 2.74	.8384
Stage (M1 vs. unresectable III)	2.09	0.66, 6.60	.2084
Elevated LDH (LDH ≥upper limit of normal)	1.002	1.001, 1.004	.0020
Elevated CD8+ TIL ≥222.9 cells/mm^2^ vs. <222.9 cells/mm^2^	0.27	0.06, 1.20	.0844
Elevated TMB ≥14.70 Mut/Mb vs. <14.70 Mut/Mb	0.45	0.18, 1.16	.0971

### Correlation Between Targeted NGS TMB/Mb, Response to Anti-PD-1 Immunotherapy, and PFS and OS

**Figure 3.**
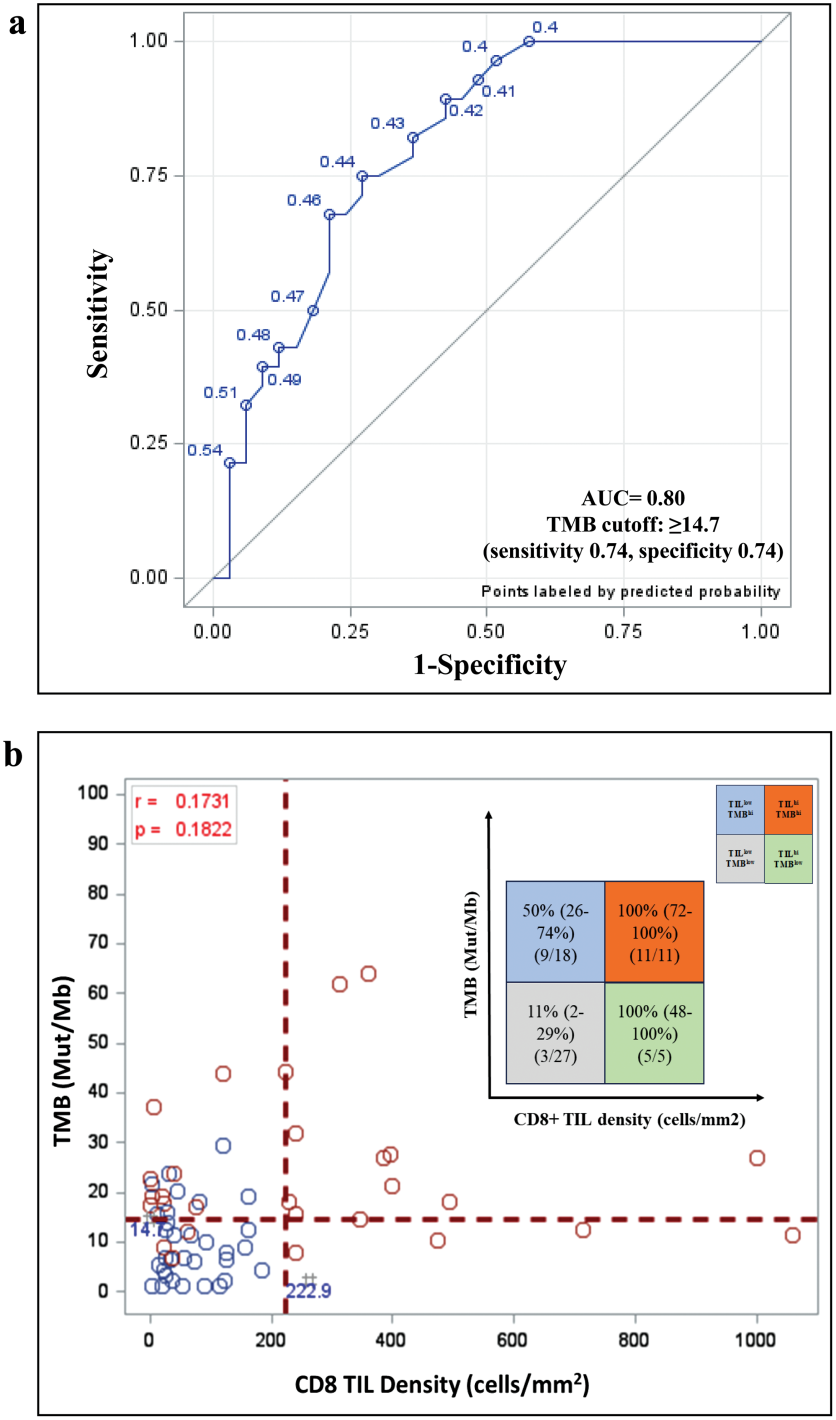
Derivation of 14.7 Mut/Mb TMB cutoff in anti-PD-1 treated melanoma patients.

**Figure 4.**
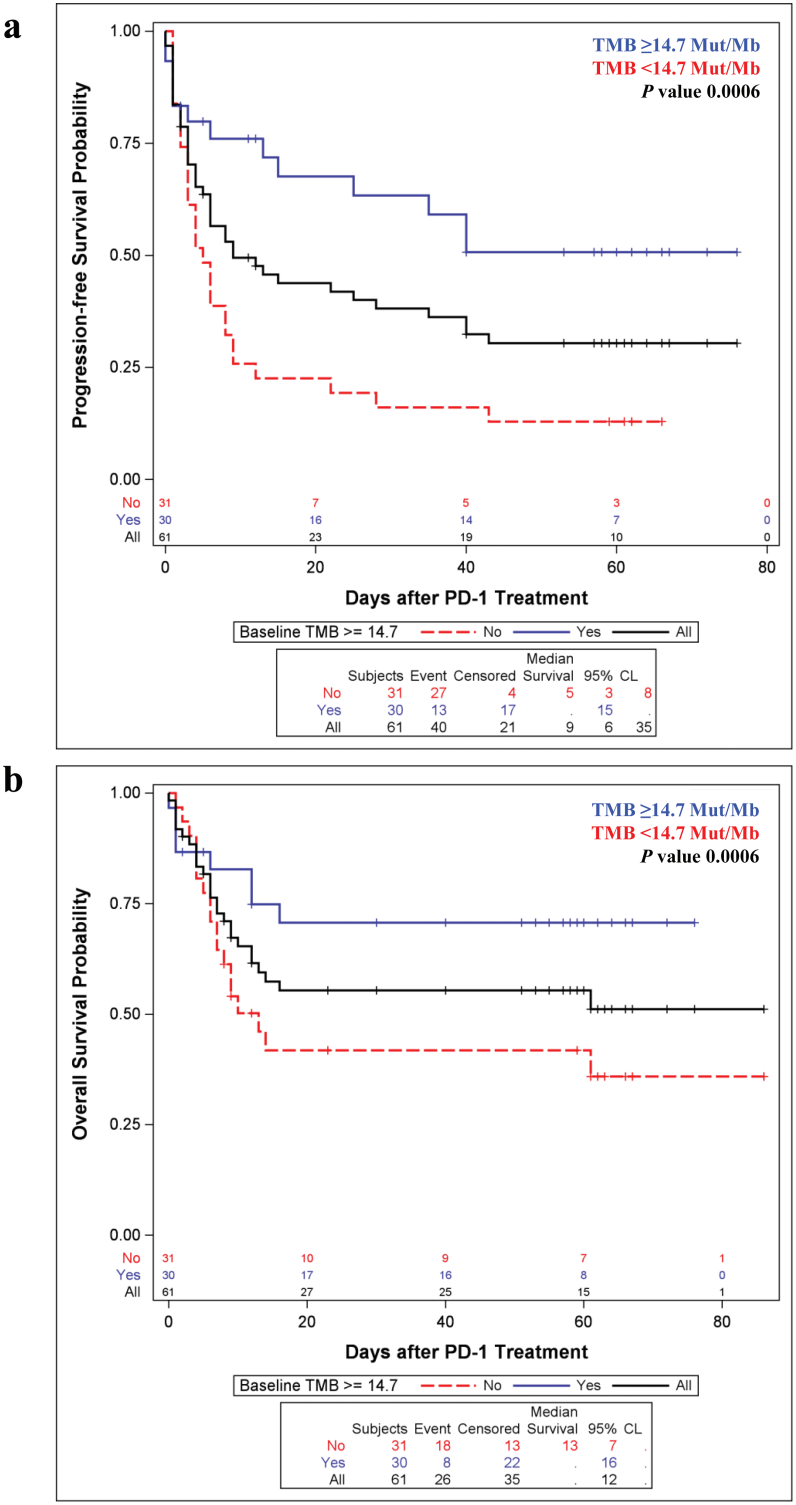
Relationship between elevated TMB and PFS and OS in PD-1 treated melanoma.

Of the seventy-eight patients in this cohort, sixty-two patients had their tumors molecularly profiled by a targeted NGS panel (see Methods). Similar to CD8+ TIL density, the TMB did not significantly differ by the biopsy source site (ANOVA *F*-test *P*-value .5346; Table 2). Consistent with prior data, increasing TMB/Mb was associated with improved response to anti-PD-1 immunotherapy. The median TMB/Mb was significantly greater in responders compared to non-responders both at 3 months (*P* = .0002), and 6 months (*P* = .0002), with one patient currently receiving therapy. Using the ROC curve method, we identified a TMB/Mb cutoff of ≥14.70 Mut/Mb which optimally segregated responders from non-responders (Fig. 3A) (sensitivity 0.74, specificity 0.74, AUC 0.80). The TMB cutoff of ≥14.70 Mut/Mb very favorably compared with that reported for melanoma patients previously.^31^ This cutoff was associated with improved odds of 3-month ORR (OR 8.0, 95% CI 2.5%-25.2%, *P* = .0004), and 6-month ORR (OR 7.9, 95% CI 2.5%-25.6%, *P* = .0006). Concordantly, TMB exceeding cutoff was significantly associated with improved PFS (*P* = .0006) and improved OS (*P* = .0305; Fig. 4). In our multivariate analysis, patients with TMB/Mb values ≥14.70 Mut/Mb continued to have significantly greater PFS than those with TMB/Mb values <14.70 Mut/Mb (*P* = .0209) with a trend toward improved OS (*P* = .0971; Table 4).

### Correlation Between CD8+ TIL Density and TMB/Mb in Melanoma Patients Receiving Anti-PD-1 Therapy

To examine how CD8+ TIL density and TMB/Mb compared to each other in predicting responsiveness to anti-PD-1 therapy, we directly compared these using a correlation scatterplot (Fig. 3B). The correlation between TMB and CD8+ TIL density was low (*r* = .1731, *P* = .1822) in this cohort (Fig. 3B). The lack of correlation, combined with the observed individual predictive values, suggests that TMB and CD8+ TIL density represent independent predictive biomarkers of response to anti-PD-1 therapy, akin to what was previously reported with TMB and T-cell–inflamed gene expression profile (GEP).^19^

## Discussion

Numerous studies have shown CD8+ TIL to be a predictive biomarker of anti-PD-1 therapy in multiple cancers^14-16^; however, there is a lack of consensus on the methods for identification and quantification of CD8+ TIL within melanoma and across tumor types between different studies.^28,29^ Given the role of varying biomarkers in the prediction of response to therapy, it would be beneficial to derive a specific, validated, and cost-effective method for analyzing CD8+ TIL within tumor types. In the context of colorectal carcinoma, the creation of a novel scoring algorithm known as the “Immunoscore” has been established as a powerful prognostic instrument for evaluating intratumoral lymphocytes based on digitally quantified densities of IHC-labeled CD3+ and CD8+ T cells.^32,33^ Furthermore, give the assay’s reproducibility across a multicenter, international study and its ability to predict disease recurrence, it has been advocated for its incorporation into the classical TNM staging (TNM-I) to provide a comprehensive prognostic assessment.^32,34^ However, the translation of this scoring algorithm to other tumor types, including melanoma, is yet to be fully studied.

Outside of melanoma, a single study in head and neck squamous cell carcinoma reported on the prognostic utility of CD8+ TIL density derived using an image analysis algorithm in patients.^28^ However, upon multivariate modeling, CD8+ TIL density retained prognostic value only in HPV-negative tumors, and further, this study did not evaluate the value of CD8+ TIL density in predicting response to immunotherapy. Within melanoma, a single study reported on the value of an automated CD8+ TIL density in predicting response to targeted therapy in BRAF mutant tumors.^29^

In this study, we report on the development of an image analysis algorithm that automatically quantified WSI CD8+ TIL density acquired from stained tissue sections from advanced melanoma patients and correlated CD8+ TIL density with response to front-line anti-PD-1 immunotherapy. Measured WSI CD8+ TIL density did not significantly differ by tissue source. A WSI CD8+ TIL density of ≥222.9 cells/mm^2^ reliably distinguished PD-1 responders from non-responders at all measured timepoints, and was associated with improved median PFS (not reached vs. 5 months) and OS (not reached vs. 16 months). WSI CD8+ TIL density was not associated with TMB, underscoring that although TMB correlates with neoantigen load,^35-37^ TIL density and TMB represented independent individual biomarkers of response to anti-PD-1, as has been independently reported.

Our study has several important limitations. The standard of care in front-line advanced melanoma in the US is one of 2 doublets (nivolumab/ipilimumab or nivolumab/relatlimab) based on improved PFS over PD-1 monotherapy on the results of pivotal trials.^38-41^ Further, in PD-1 naïve BRAF mutant melanoma, initial treatment with nivolumab/ipilimumab followed by targeted therapy in the event of progression is associated with superior OS, compared to the reverse sequence.^42^ Based on this, combination immunotherapy as the initial therapy of choice for most advanced melanoma patients in the US. However, as we required sufficient post-treatment follow-up to infer survival endpoints, we limited our cohort to PD-1 monotherapy treated patients with > 3 years follow-up time. As such, it remains unclear how this biomarker will discriminate between responders and non-responders to nivolumab/relatlimab or nivolumab/ipilimumab administered in the front line setting. Further, the sample size was small, not powered a priori for biomarker evaluation, nor has this score been evaluated in an independently treated validation cohort from a different institution.

## Conclusion

To our knowledge, our study is the first within melanoma that has evaluated a digital analysis algorithm to quantify WSI CD8+ TIL density and derived a predictive value associated with response to front-line anti-PD-1 therapy in a real-world setting. The value of a predictive biomarker to PD-1 monotherapy is considerable, given the magnitude of benefit observed in biomarker-high vs. biomarker-low patients in terms of response (6-month ORR 94.7% vs. 21.1%), median PFS (not reached vs. 5 months) and median OS (not reached vs. 16 months). We additionally show that CD8+ TIL density is independent of TMB in predicting front-line anti-PD-1 response, suggesting that these 2 biomarkers capture distinct features of neoantigenicity and T-cell activation in inflamed tumors such as melanoma. The validation of this in independent cohorts of melanoma patients treated with combination immunotherapy is ongoing.

## Supplementary Material

oyae054_suppl_Supplementary_Tables_1

## Data Availability

The datasets used and/or analyzed during the current study are available from the corresponding author on reasonable request.
