# Supplementary material for: Automated Quantitative CD8+ Tumor-Infiltrating Lymphocytes and Tumor Mutation Burden as Independent Biomarkers in Melanoma Patients Receiving Front-Line Anti-PD-1 Immunotherapy
Source: Oncologist. 2024 Apr 24;29(7):619–28. doi: 10.1093/oncolo/oyae054 (PMC11224974; doi:10.1093/oncolo/oyae054)
Supplement: oyae054_suppl_Supplementary_Tables_1 [file oyae054_suppl_supplementary_tables_1.docx]

**SUPPLEMENTARY TABLES**

**Supplementary Table 1A: List of Assayed Genes**

| **Hotspot Genes** | | | | **Full-length Genes** | | | **Copy Number Genes** | | |
| --- | --- | --- | --- | --- | --- | --- | --- | --- | --- |
| *AKT1* | *ESR* | *KIT* | *PI3KCA* | *ARID1A* | *FBXW7* | *PTEN* | *AKT1* | *FGFR2* | *TERT* |
| *AKT2* | *EZH2* | *KNSTRN* | *PI3KCB* | *ATM* | *MLH1* | *RAD50* | *AKT2* | *FGFR3* | *TSC1* |
| *AKT3* | *FGFR1* | *KRAS* | *PPP2R1A* | *ATR* | *MRE11A* | *RAD51* | *AKT3* | *FGFR4* | *TSC2* |
| *ALK* | *FGFR2* | *MAGOH* | *PTPN11* | *ATRX* | *MSH2* | *RAD51B* | *ALK* | *FLT3* |  |
| *AR* | *FGFR3* | *MAP2K1* | *RAC1* | *BAP1* | *MSH6* | *RAD51C* | *AR* | *IGF1R* |  |
| *ARAF* | *FGFR4* | *MAP2K2* | *RAF1* | *BRCA1* | *NBN* | *RAD51D* | *AXL* | *KIT* |  |
| *AXL* | *FLT3* | *MAP2K4* | *RET* | *BRCA2* | *NF1* | *RB1* | *BRAF* | *KRAS* |  |
| *BRAF* | *FOXL2* | *MAPK1* | *RHEB* | *CDK12* | *NF2* | *RNF43* | *CCND1* | *MDM2* |  |
| *BTK* | *GATA2* | *MAX* | *RHOA* | *CDKN1B* | *NOTCH1* | *SETD2* | *CCND2* | *MDM4* |  |
| *CBL* | *GNA11* | *MDM4* | *ROS1* | *CDKN2A* | *NOTCH2* | *SLX4* | *CCND3* | *MET* |  |
| *CCND1* | *GNAQ* | *MED12* | *SF3B1* | *CDKN2B* | *NOTCH3* | *SMARCA4* | *CCNE1* | *MYC* |  |
| *CDK4* | *GNAS* | *MET* | *SMAD4* | *CHEK1* | *PALB2* | *CMARCB1* | *CDK2* | *MYCL* |  |
| *CDK6* | *HSF3A* | *MTOR* | *SMO* | *CREBBP* | *PIK3R1* | *STK11* | *CDK4* | *MYCN* |  |
| *CHEK2* | *HIST1H3B* | *MYC* | *SPOP* | *FANCA* | *PMS2* | *TP53* | *CDK6* | *NTRK1* |  |
| *CSF1R* | *HNF1A* | *MYCN* | *SRC* | *FANCD2* | *POLE* | *TSC1* | *CDKN2A* | *NTRK2* |  |
| *CTNNB1* | *HRAS* | *MYD88* | *STAT3* | *FANCI* | *PTCH1* | *TSC2* | *CDKN2B* | *NTRK3* |  |
| *DDR2* | *IDH1* | *NFE2L2* | *TERT* |  |  |  | *EGFR* | *PDGFRA* |  |
| *EGFR* | *IDH2* | *NRAS* | *TOP1* |  |  |  | *ERBB2* | *PDGFRB* |  |
| *ERBB2* | *JAK1* | *NTRK1* | *U2AF1* |  |  |  | *ESR1* | *PIK3CA* |  |
| *ERBB3* | *JAK2* | *NTRK2* | *XPO1* |  |  |  | *FGF19* | *PIK3CB* |  |
| *ERBB4* | *JAK3* | *PDGFRA* |  |  |  |  | *FGF3* | *PPARG* |  |
| *ERCC2* | *KDR* | *PDGFRB* |  |  |  |  | *FGFR1* | *RICTOR* |  |

**Supplementary Table 1B: List of Gene Fusions (inter- and intra- genic) Included in Oncomine Pan-Cancer Panel**

| **Gene Fusions (inter- and intra- genic)** | | |
| --- | --- | --- |
| *AKT2* | *FGFR2* | *NUTM1* |
| *ALK* | *FGFR3* | *PDGFRA* |
| *AR* | *FGR* | *PDGFRB* |
| *AXL* | *FLT3* | *PIK3CA* |
| *BRAF* | *JAK2* | *PPARG* |
| *BRCA1* | *KRAS* | *PRKACA* |
| *BRCA2* | *MDM4* | *PRKACB* |
| *CDKN2A* | *MET* | *PTEN* |
| *EGFR* | *MYB* | *RAD51B* |
| *ERBB2* | *MYBL1* | *RAF1* |
| *ERBB4* | *NF1* | *RB1* |
| *ERG* | *NOTCH1* | *RELA* |
| *ESR1* | *NOTCH4* | *RET* |
| *ETV1* | *NRG1* | *ROS1* |
| *ETV4* | *NTRK1* | *RSPO2* |
| *ETV5* | *NTRK2* | *RSPO3* |
| *FGFR1* | *NTRK3* | *TERT* |
| Note: While the Oncomine Pan-Cancer Panel includes this list of inter- and intra- genic fusions, as we only analyzed the DNA portion of the assay to evaluate for TMB, these genes were not covered during this analysis. | | |
